# A Response Surface Methodology Study for *Chlorella vulgaris* Mixotrophic Culture Optimization

**DOI:** 10.3390/microorganisms12020379

**Published:** 2024-02-12

**Authors:** Sandra Milena Rincon, Haluk Beyenal, Hernán Mauricio Romero

**Affiliations:** 1 The Gene and Linda Voiland School of Chemical Engineering and Bioengineering, Washington State University, Pullman, WA 99163, USA; srinconmiranda@gmail.com (S.M.R.); beyenal@wsu.edu (H.B.); 2 Biology and Breeding Research Program, Colombian OiI Palm Research Center, Bogotá 111121, Colombia; 3 Department of Biology, Universidad Nacional de Colombia, Bogotá 11132, Colombia

**Keywords:** magnesium concentration, response surface methodology, mixotrophic cultivation, urea, glycerol, fatty acid profile

## Abstract

Glycerol is a carbon source that produces good biomass under mixotrophic conditions. Enhancing the composition of culture media in algae biomass production improves growth rates, biomass yield, nutrient utilization efficiency, and overall cost-effectiveness. Among the key nutrients in the medium, nitrogen plays a pivotal role. Urea can be effectively used as a nitrogen source and is considered a low-cost form of nitrogen compared to other sources. Urea metabolism releases some CO_2_ in photosynthesis, and magnesium plays a major role in urea uptake. Magnesium is another key nutrient that is key in photosynthesis and other metabolic reactions. To maximize glycerol consumption in the mixotrophic system and to obtain high biomass and lipid productions, the variations in MgSO4·7H_2_O and urea concentrations were evaluated in the growth medium of the microalgae. A response surface methodology (RSM) using a central composite design (CCD) was designed to maximize glycerol consumption at the initial cellular growth rates (up to four days). The magnesium and urea supply varied from 0.3 to 1.7 g L^−1^. Response surface methodology was utilized to analyze the results, and the highest glycerol consumption rate, 770.2 mg L^−1^ d^−1^, was observed when *C. vulgaris* was grown at 1.7 g L^−1^ urea, 1.0 g L^−1^ MgSO4·7H_2_O. Using the optimal urea and magnesium concentrations with acetate, glucose, and glycerol as carbon sources, the same lipid content (10% average) was achieved on day 4 of mixotrophic *C. vulgaris* culture. Overall, the results show that mixotrophic growth of *C. vulgaris* using urea with an optimum magnesium concentration yields large amounts of fatty acids and that the carbon source greatly influences the profile of the fatty acids.

## 1. Introduction

The metabolic processes that regulate microalgal growth depend on factors such as pH, temperature, illumination, and nutrient availability. Depending on these conditions, microalgae can grow autotrophically, heterotrophically, or mixotrophically. Mixotrophic algae have shown remarkable nutrient assimilation with high biomass and oil production productivity [1,2,3]. This mode of cultivation requires carbon and nitrogen in the medium for microalgae biomass accumulation in the presence of light. Thus, to scale up the mixotrophic culture, it is necessary to use cost-effective nutrient sources, i.e., low-cost nitrogen and organic carbon substrates. Possible sources include urea, a cheap nitrogen source supplied as fertilizer in agriculture, and glycerol, a carbon source coproduct of the biodiesel industry [4,5].

Enhancing the composition of culture media in algae biomass production improves growth rates, biomass yield, nutrient utilization efficiency, and overall cost-effectiveness. This optimization is a pivotal factor in the success of algal cultivation across diverse applications. Achieving efficient and effective optimization of algae culture is feasible through the design of experiments utilizing statistical models. One widely utilized statistical model type is response surface methodology (RSM). These models explore a system’s response to various combinations of variables, tailoring the approach to the algae cultivation system’s specific goals, constraints, and characteristics. Thus, the microalga *Tetradesmus obliquus* achieved a remarkable 32% increase in biomass yield, attributed to a well-balanced combination of essential nutrients such as urea, potash, superphosphate, and magnesium sulfate. This optimized nutrient mix, developed through the Central Composite Rotary Design (CCRD), outperformed the commonly employed N 11 medium in a raceway pond setting. The cost of production was substantially reduced by 47-fold, thanks to the utilization of an economical growth medium formulated with locally available agricultural fertilizers, replacing the more costly analytical grade inorganic salts. This approach showcased superior biomass yields per unit of time and resources [6]. Furthermore, customizing the culture media for *Chlorella sorokiniana* in outdoor photoautotrophic conditions resulted in accelerated growth rates, achieving a biomass of 3.58 ± 0.07 gL^−1^. Additionally, the yields of polysaccharides and proteins surpassed 1.5 times the baseline, aligning with the specific nutritional requirements for nitrogen (urea), carbon (Na_2_CO_3_), ventilation capacity (40 L h^−1^), and magnesium [7]. In another study, the impact of MgO nanoparticles on lipid accumulation in the indigenous microalga *Desmodesmus* sp. *VV2* was investigated using RSM, and the findings were further confirmed through validation using an artificial neural network (ANN) employing 11 training algorithms. The optimization process, involving CaCl_2_, NaNO_3_, and MgO, resulted in a lipid content of 57.6%. The study indicated that RSM exhibited greater significance than ANN [8]. In summary, through meticulous adjustment of the culture media, it becomes feasible to reduce the overall production costs by minimizing the usage of costly nutrients and supplements while still attaining optimal growth and lipid and biomass yield.

Urea is the nitrogen source with the highest percentage of nitrogen by weight available (46.7% nitrogen). Urea is metabolized to ammonia and carbon dioxide inside the cell through a sequential assimilation mechanism. First, urea enters the microalgae cells via either passive or active transport [9]. Second, urea assimilation in *Chlorella* spp. and *Chlamydomonas* spp., which lack the urease enzyme [10,11], follows two sequential reactions. The first reaction requires adenosine triphosphate (ATP) in the presence of Mg^2+^, a univalent cation such as Na^+^, K^+^, or NH_4_^+^, and bicarbonate. The urea carboxylase catalyzes the ATP-dependent carboxylation of urea to allophanate (Equation (1)). In the second reaction, the allophanate is subsequently hydrolyzed to ammonia and carbon dioxide by allophanate hydrolase (Equation (2)) [9,11,12,13].

(1) $$CO-{\left(NH_{2}\right)}_{2}+{{HCO}_{3}}^{-}+ATP\;\to \;(NH_{2})-CO-NH-{COO}^{-}+H_{2}O+ADP+Pi$$

(2) $$(NH_{2})-CO-NH-{COO}^{-}+3H_{2}O+H^{+}\to \;{2NH_{4}}^{+}+{2HCO_{3}}^{-}$$

In this two-step assimilation mechanism of urea, Mg^2+^ is a crucial cofactor that may be determinant for the rate of CO_2_ production at the end of the two reactions. Mg^2+^ is also critical in the physiological and biochemical processes of photosynthetic organisms. For instance, Mg^2+^ activates ribulose-1,5-bisphosphate (RuBP) carboxylase, the key enzyme in photosynthesis. Also, Mg^2+^ is essential for light absorption because it is the central element in chlorophyll. Furthermore, Mg^2+^ acts as a cofactor of enzymes involved in CO_2_ fixation and is involved in energy transfer via ATP and in pH control [14]. Moreover, Mg^2+^ participates in glycolysis, the Krebs cycle, and ion transport across cell membranes [15]. As a result, Mg^2+^ is a fundamental component in our developed mixotrophic system, in which CO_2_ and O_2_ production are internally balanced for the autotrophic and heterotrophic metabolisms to work [16].

In the mixotrophic metabolism of microalgae, an external carbon source is required. Organic carbon sources such as glucose and glycerol have been used [1,17,18,19], with glycerol gaining relevance due to several characteristics. Glycerol is mostly obtained as a by-product during biodiesel production via transesterification, and it can be a source of carbon and energy for microalgal growth [20]. In the cytoplasm, glycerol is converted into glyceraldehyde 3-phosphate (G3P), intersecting several metabolic pathways, such as glycolysis and gluconeogenesis. The G3P can be converted into lipids, carbohydrates, or amino acids. The G3P may also be further converted into pyruvate, which can be oxidized by the TCA cycle within the mitochondrion for energy generation [20,21].

Several studies have examined the growth rates and total lipid contents of various microalgal species supported by glycerol [17,22,23,24] (Table 1). These studies’ findings have shown improvements in mass culture productivity compared to phototrophic cultures. Notably, adding glycerol causes a 2.4-fold increase in growth rate, up to a 1.9-fold increase in biomass, and a 40–60% increase in total lipid production [25].

The CO_2_ fixed into the biomass from glycerol and urea in *C. vulgaris* mixotrophic cultures can be consumed via the Calvin–Benson cycle using light energy [26]. Here, the CO_2_ is released into the medium by the glycerol consumed heterotrophically for energy and by urea hydrolysis. Thus, there is a synergistic gas exchange under mixotrophic cultivation in which the O_2_ from photosynthesis is consumed in the catabolism of glycerol. In contrast, the liberated CO_2_ from urea metabolism and glycerol respiration can be used in photosynthesis [16]. Consequently, the growth of *Chlorella vulgaris* in this mixotrophic system without external CO_2_ supply requires a high consumption of glycerol, which is highly dependent on the optimal urea metabolism, not only for consumption as a nitrogen source but also for autotrophic release of O_2_.

To maximize glycerol consumption in the mixotrophic system and to obtain high biomass and lipid production, the variations in MgSO_4_·7H_2_O and urea concentrations were evaluated in the growth medium of the microalgae. A response surface methodology (RSM) using a central composite design (CCD) was designed to maximize glycerol consumption at the initial cellular growth rates (up to four days). Once the optimum growth medium for mixotrophic *C. vulgaris* growth was obtained, other carbon sources were tested to determine the differences in fatty acid profiles and nitrogen and phosphorus removal. We compared *C. vulgaris* performance using various carbon sources (glycerol, glucose, sodium acetate) widely used in microalgae mixotrophic systems [27].

**Table 1:** Biomass and lipid production of *C. vulgaris* using different concentrations of glycerol, magnesium, and nitrogen in mixotrophic cultivation.

Glycerol Concentration (g L^−1^)	NaNO_3_ (mg L^−1^)	MgSO_4_·7H_2_O (mg L^−1^)	Biomass Concentration (g L^−1^)	Biomass Productivity (g L^−1^ d^−1^)	Lipid Content (%)	Lipid Productivity (g L^−1^ d^−1^)	Specific Growth Rate (d^−1^)	Ref.
2.7	750	75	1.56	0.17 ± 0.03			0.280 ± 0.09	[28]
2	1500	75	1.50	0.23 ± 0.02	15.91 ± 1.50		0.342 ± 0.03	[29]
20	250	75	0.66 ± 0.01	0.09 ± 0.01	34 ± 4.00	0.031 ± 0.004		[30]
20.4	1000	500	1.17 ± 1.34	0.39 ± 0.45	40.10 ± 22.06	0.16 ± 0.10		[31]
5	800	50	1.91	0.227 ± 0.007	15.91%		0.342 ± 0.004	[22]
5	250	75	2.13 ± 0.34	0.53 ± 0.08			0.94 ± 0.04	[17]
4	100	400	2.64 ± 0.22	0.19 ± 0.01	20.36 ± 5.30	0.43 ± 0.09		[32]
10	Soil extract		1.32 ± 0.27		26.90 ± 0.21			[23]

The goals of our work were (1) to assess the effects of the magnesium and urea concentrations on the maximum glycerol consumption rate when glycerol concentration and light intensity were not limiting and (2) to compare the oil composition and the nitrogen and phosphorus removal with acetate, glucose, and glycerol as carbon sources at the optimal magnesium and urea concentrations determined for mixotrophic growth. To achieve our goals, *C. vulgaris* was cultured mixotrophically in a planktonic system.

## 2. Materials and Methods

### 2.1. Chlorella vulgaris Growth Conditions

Green alga *Chlorella vulgaris* Beijerinck (UTEX 29) was obtained from the American Type Culture Collection (ATCC^®^ 30,581™). The M8A growth medium consisted of 1.0 to 1.8 g L^−1^ CO (NH_2_)_2_ (variable), 0.3 to 1.0 (variable) g L^−1^ MgSO_4_·7H_2_O, 1.48 g L^−1^ KH_2_PO_4_, 0.52 g L^−1^ Na_2_HPO_4_·2H_2_O, 0.013 g L^−1^ CaCl_2_·2H_2_O, 0.116 g L^−1^ EDTA ferric sodium salt, 0.0372 g L^−1^, Na_2_EDTA·2H_2_O, 6.18·10^−5^ g L^−1^ H_3_BO_3_, 0.013 g L^−1^ MnCl_2_·4H_2_O, 3.2·10^−3^ g L^−1^ ZnSO_4_·2H_2_O, and 1.83·10^−3^ g L^−1^ CuSO_4_ 5H_2_O.

All mixotrophic cultivations of *C. vulgaris* were performed in 50-mL Erlenmeyer flasks containing 15 mL of liquid medium. The stock solutions were individually sterilized, and the media were prepared directly in flasks under a hood. The pH was adjusted to 7.5 before the liquid media were inoculated with 0.002 g of algae inoculums. Changes in pH were not monitored or controlled during the experiment (4 days). The Erlenmeyer flask was kept on a shaker (Unimax 2010, Heidolph, Schwabach, Germany) at 130 revolutions per minute (rpm) and incubated in a controlled environment chamber (Fitotron^®^ SGC 120, Weiss Technik Inc., Grand Rapids, MI, USA) at a maximum of 80 µmol photons m^−2^ s^−1^ for a 14-h photoperiod for 4 days. The temperature inside the chamber was set at 28 ± 1 °C. The glycerol, acetate, and glucose were sterilized for the essays with various organic carbon sources. All experiments were biologically replicated at least four times.

### 2.2. Experimental Design and Statistical Analysis

Response surface methodology (RSM) coupled with central composite design (CCD) was employed to optimize the mixotrophic *C. vulgaris* planktonic growth and assess the effects of the magnesium and urea concentrations on the maximum glycerol consumption rate. Two independent variables, magnesium (in the form of MgSO_4_·7H_2_O) and urea concentrations, were varied in this study over three levels between −1 and +1, in the determined ranges based on a set of preliminary experiments. The ranges of these variables were based on previous results obtained at pH 6, 7, and 8, in which the urea and MgSO_4_·7H_2_O concentrations were at least triple those used here. Thirteen CCD experiments were performed with 5 replications to assess the pure error. The total number of experiments in CCD was defined by $n=2^{k}+2k+n_{0}$, where *k* is the number of factors. The 13 sets of experiments correspond to four edge points (factorial points), four star points (at some distance from the center point at α ± 1), and five replicates at the central point (n_0_). The axial parameter (α) is $\alpha =f^{\frac{1}{4}}$, where F = 4 center points [33]. XLSTAT software (Version 19.6, 32 bit, Addinsoft, Paris, France) was used to design the experiments and analyze the data. The obtained experimental data were fitted to the following quadratic polynomial equation:

(3) $${Y=\beta }_{0}+\sum_{i=1}^{k}\beta_{i}x_{i}+\sum_{i=1}^{k}\beta_{ii}{x_{i}}^{2}+\sum_{i<j}\sum \beta_{ij}x_{i}x_{j}+\varepsilon$$

where *Y* is the response variable; *β* is a vector of *p* unknown constant coefficients (*β*_0_ is the constant coefficient; *βi*, *βii*, and *βij* are the coefficients for the linear, quadratic, and interaction effects), *xi* and *xj* are the coded variables, and *ε* is a random experimental error assumed to have a zero mean. The correlation coefficient (R^2^) was quantified using the same program to estimate the fit to the polynomial model. Analysis of variance (ANOVA) was used to analyze the data to obtain the interaction between the variables and the responses, and the F test was used to check its statistical significance.

### 2.3. Effects of Organic Carbon Sources on C. vulgaris Nitrogen and Phosphorus Removal and Fatty Acid Profile

These essays were run under the cultivation conditions explained in the previous section. Three organic carbon sources were used: acetate, glucose, and glycerol. The concentration of carbon in the media was set according to C-equivalents to supply the same amount of carbon to the liquid media. Thus, multiplying the C-equivalents of glycerol by a factor of three, the concentrations used were 54 mM glycerol (3 C-equivalents), 27 mM glucose (6 C-equivalents), and 81 mM acetate (2 C-equivalents). All experiments were conducted in four biological replicates. The carbon concentration used here is within the range previously reported for *C. vulgaris* growth.

The liquid samples were taken at the end of the experiment (4 days) after the media were filtered through a 0.45-mm filter. These samples were analyzed for total nitrogen and phosphorus with a segmented continuous flow (CFA) automated equipment (QuAAtro CFA systems, Seal Analytica, Mequon, WI, USA). Based on the standard procedure [34,35], phosphorus (P) and nitrogen (N) were detected colorimetrically at 520 nm and 880 nm, respectively. The removal rate was calculated as the concentration difference between the experiment start and end for total−N and total−P in the culture supernatant.

The total lipid content of *C. vulgaris* was extracted using a chloroform–methano solution (Folch solvent) and the conventional solvent extraction method. Fatty acid methyl esters (FAMEs) were prepared as follows: 100 mg of oil was saponified with 2 mL of KOH/MeOH 2N for 10 min at 70 °C with stirring. After this, the free fatty acids were derivatized to FAMEs by adding 3 mL of BF_3_/MeOH (20% Merck, Newark, NJ, USA) to the previous solution, which was slightly cooled. Finally, FAMEs were extracted with 2 mL of hexane. FAMEs were analyzed using a gas chromatograph (7890A, Agilent Technologies, Santa Clara, CA, USA) equipped with a flame ionization detector (FID) and split/splitless automatic injector. A fused silica capillary column (60 m × 0.25 mm ID × 0.25 µm film thickness. Agilent J&W DB-23, Agilent Technologies, Santa Clara, CA, USA) was used. The oven temperature was programmed as follows: 50 °C for 1 min, next increased to 175 °C at 25 °C min^−1^, then increased to 230 °C at 4 °C min^−1^, and kept at 230 °C for 7 min. The injection and detector temperatures were 250 °C. The carrier gas was hydrogen at 33 cm s^−1^ at 50 °C. The split ratio was 50:1. FAME identification was based on retention times compared with the standard (Supelco 37-component FAME Mix, Cat. 47885-U, Supelco, Newark, NJ, USA).

### 2.4. Urea and Glycerol Concentration Measurements

Samples of bulk medium were taken at the end of four consecutive days of *C. vulgaris* mixotrophic growth. The concentration was measured by HPLC using an Agilent AT6890A Series Plus chromatograph equipped with a Zorbax SB-C18 (150 × 4.6 mm, 3.5 um) column (Agilent Technologies, Santa Clara, CA, USA). In urea quantification, the temperature was constantly held at 35 °C. The mobile phase of two eluents, (A) ACN and (B) H_2_O, was programmed in gradient elution as follows: 20% A (0.6 min), 60% A (12.6 min), 100% A (13.6 min), and 100% A (20.6 min) at a constant flow rate of 0.5 mL min^−1^. The HPLC auto-sampler was programmed to perform xanthydrol derivatization as described by Clark et al. [36] with two modifications in the procedure: (1) mixture in the air at a maximum speed seven times instead of three times and (2) a waiting period of 6 min instead of 5 min. The derivatized analyte was detected with excitation and emission wavelengths of 213 nm and 308 nm, respectively. The glycerol concentration was determined according to Frieler et al. [37] using benzoyl chloride as the derivatization reagent. The HPLC separation was performed at a constant temperature of 55 °C. The flow rate of the mobile phase was 1 mL min^−1^. The gradient elution program was modified as follows: (1) ACN; (2) H_2_O: 30% A (0 min), 50% A (20 min), 80% A (25 min), 80% A (27 min). The derivatized analyte was detected with a UV detector at 231 nm.

The consumption rates (CR; mg L^−1^ day^−1^) of urea and glycerol were calculated as follows: CR = (Ci − Cf)/t, where Ci and Cf are the nutrient concentrations (mg L^−1^) at the beginning and end of the growth cycle, respectively, and t is the time interval (days).

## 3. Results

### 3.1. Regression Model and Statistical Analysis

The results of the 13 runs of the CCD experimental design are shown in Table 2. The observed glycerol consumption rate varied between 276.5 and 770.2 mg L^−1^ d^−1^. The highest glycerol consumption rate was obtained in run 6, at a urea medium concentration of 1.7 g L^−1^ and an MgSO_4_·7H_2_O concentration of 1.0 g L^−1^ for mixotrophic *C. vulgaris* planktonic growth. The second-order polynomial equation obtained by multiple regression analysis is represented below:

$$Glycerol\;consumption\;rate=525.3+95.56*Urea+5.51*Mg+29.84*{Urea}^{2}\;-74.24*{Mg}^{2}+167.82*Urea*Mg$$

The statistical significance of the equation was evaluated by F-test and ANOVA, which showed that the model was statistically significant at a 95% confidence level (*p* < 0.05) (Table 3). The model also showed that the lack of the first parameter was statistically nonsignificant (*p* > 0.05), indicating that the response was adequate for use in this model (see Table 3). The model’s coefficient of determination (R^2^) was 0.913, and the adjusted R^2^ was 0.851, indicating that the model adequately represents the relationships among the selected reaction variables. It also indicates that the response model can explain 91.3% of the variability.

The *p*-values lower than 0.05 indicate which model terms are significant. In this case, the urea concentration, the magnesium quadratic term, and the interaction between the urea and magnesium concentrations were significant model terms.

**Figure 1:**
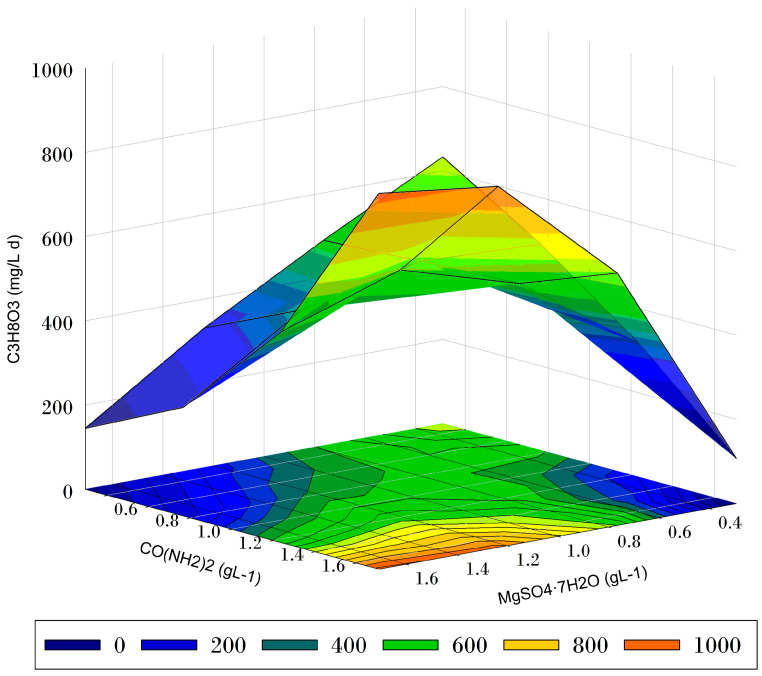
Three-dimensional response surface and the corresponding contour plot for the CH_3_H_8_O_3_ (glycerol) consumption rate after four days of mixotrophic *C. vulgaris* planktonic growth at the initial MgSO_4_·7H_2_O and CO (NH_2_)_2_ (urea) concentrations. The black circles are the experimental data.

The three-dimensional (3D) response surface and contour plot in Figure 1 shows the effects of the experimental variables, the urea and MgSO_4_·7H_2_O concentrations, on the response of glycerol consumption rate after four days of mixotrophic *C. vulgaris* planktonic growth. Urea concentrations higher than 1.5 g L^−1^ and MgSO_4_·7H_2_O concentrations higher than 1.0 g L^−1^ improved glycerol consumption rates. In contrast, a decline in the glycerol consumption rate resulted from a MgSO_4_·7H_2_O concentration below 0.6 g L^−1^ or a urea concentration below 1.0 g L^−1^.

A test was performed with the point-predicted solution of mixotrophic *C. vulgaris* planktonic growth (at 1.5 g L^−1^ urea and 1.0 g L^−1^ MgSO_4_·7H_2_O) to confirm the adequacy and validity of the optimization procedure. The experimental values matched well with their predicted counterparts (Table 4).

### 3.2. The Fatty Acid Composition Obtained in C. vulgaris Cultured with Glycerol, Acetate, and Glucose

**Figure 2:**
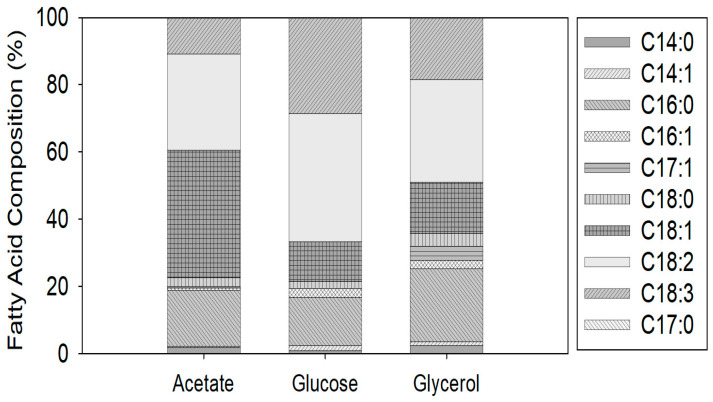
Fatty acid composition obtained in *C. vulgaris* cultures with glycerol, acetate, or glucose at four days under mixotrophic conditions with urea as the nitrogen source.

At the optimal urea and initial magnesium concentrations (1.7 g L*^−^*^1^ of urea and 1.0 g L*^−^*^1^ of MgSO_4_·7H_2_O) for glycerol consumption in the mixotrophic growth of *C. vulgaris*, the total lipid content was 10% of dry weight (DW) on average for the various carbon sources evaluated (Table 5). However, the carbon source significantly influenced the fatty acid profile (Figure 2). Palmitic acid (C16:0) was the primary saturated fatty acid in all cases, with a total saturated content of 21.11% for acetate, 17.26% for glucose, and 27.82% for glycerol. Oleic acid (C18:1) was the most abundant unsaturated fatty acid in acetate culture, with 38% of the total, while linoleic acid (C18:2) was the most abundant in glucose and glycerol cultures, with 38% and 30%, respectively. The unsaturated fatty acid contents were 78.89% acetate, 82.73% glucose, and 72.17% glycerol. The contents of stearic acid (C18:0) varied slightly, while the contents of unsaturated γ-linolenic acid (C18:3) varied markedly among samples.

### 3.3. Nitrogen and Phosphorus Removal by C. vulgaris for Various Organic Carbon Sources

The effects of the organic carbon source (glycerol, acetate, or glucose) on the urea, nitrogen, and phosphorus consumption rates of *C. vulgaris* are shown in Table 5. The final pH value was reduced in the presence of glucose and glycerol, while the final pH was augmented in the presence of acetate. Another observed difference was that the phosphorus was consumed by *C. vulgaris* at a higher rate when acetate and glycerol were supplied compared with glucose carbon sources. The urea consumption rate differed strikingly for all organic carbon sources evaluated. When glycerol was used as the carbon source, the nitrogen rate reached its highest value among carbon sources.

## 4. Discussion

*C. vulgaris* produced a dry biomass concentration of 0.085 g DW in four days of growth in the growth media with 5 g L^−1^ glycerol, and the optimal levels of urea and MgSO_4_·7H_2_O, obtained using a response surface methodology analysis (1.7 g L^−1^ urea and 1.0 g L^−1^ MgSO_4_·7H_2_O). Under these conditions, the glycerol consumed was 3.8 g L^−1,^ and the urea consumed was 0.89 g L^−1^. The biomass productivity reached 1.4 g L^−1^ d^−1^, the biomass concentration was 5.59 g L^−1^, and the growth rate varied from 0.75 to 0.96 d^−1^. The statistical analysis indicated that Mg^+2^ significantly promotes the growth of *C. vulgaris* when the carbon and nitrogen sources are glycerol and urea (*p* = 0.0105 for the quadratic term in the model) (Table 3). As shown in Figure 1, the glycerol consumption rate was strongly influenced by adding both urea and MgSO_4_·7H_2_O (ANOVA F = 14.7, degrees of freedom = 5, *p* = 0.0014).

The productivity parameters obtained here were higher than those found in previous studies with glycerol in the mixotrophic culture of *Chlorella* spp. (Table 1). The present study differs fundamentally from the others in that the nitrogen source here was urea, whereas other studies used KNO_3_ for the mixotrophic growth [28,29,30,31,32]. Interestingly, mixotrophic *C. vulgaris* cultured with glucose (at 15 g L^−1^, which is equivalent to 7.7 g L^−1^ glycerol) and KNO_3_ (1 g L^−1^) but without urea showed 4.85 g L^−1^ biomass concentration, 1.62 g L^−1^ d^−1^ biomass accumulation, and 15.43% lipid content after 72 h of growth [31], which are productivity parameters comparable with those obtained in this research. Also, magnesium concentrations in previous studies (Table 1) were below the optimal concentration found here (1 g L^−1^). Cultivation with urea results in better *C. vulgaris* growth than KNO_3_ when glycerol is the carbon source.

After four days of *C. vulgaris* growth, the final pH varied among the organic carbon sources tested (glycerol, acetate, and glucose). The results showed that during *C. vulgaris* growth, there was acidification of the medium supplemented with glucose, whereas alkalization was present with acetate (Table 5). In microalgal cells, nitrogen can be metabolized by various mechanisms, and the redox regulation is different in each one of them [38]. Even though the medium was prepared with phosphate buffer pH 7.5 and urea, during the microalgae growth, the pH was relatively stable [11,39], and ionic charges were present in the acetate- and glucose-supplemented media but not in the medium supplemented with glycerol. Thus, it is plausible that the CO_2_ availability in the medium was higher than other carbon sources in the presence of glycerol. Considering that similar C-equivalents were used in the various carbon sources tested in this experiment, there may be a high level of interaction between photosynthesis and respiration under glycerol cultivation that reduces the shift in pH.

Cultivating *C. vulgaris* using acetate, glucose, and glycerol reached biomass production rates of 1.03, 0.7, and 0.88 g L^−1^ d^−1^, respectively. These rates were achieved by incorporating 1.7 g L^−1^ urea and 1.0 g L^−1^ MgSO_4_·7H_2_O in the growth media under a light intensity of 80 µmol photons m^−2^ s^−1^. As anticipated, the observed biomass accumulation exhibited no significant variation across the diverse carbon sources employed, primarily due to the same supply of C-equivalents in the media.

In this study, the conversion of urea was similar (65% on average) for all carbon sources tested. Even though *C. vulgaris* cells can use urea as an additional carbon source [11,40], the differences in N removal found among the tested carbon sources (36% for glycerol, 9% for acetate, and 7% for glucose) suggest limited ammonium assimilation from the medium by the microalgae (see Equations (1) and (2)) regardless of the carbon source.

Furthermore, it can be inferred that NH_4_^+^ removal was higher in glycerol than in acetate or glucose because the pH level was within the optimal growth range for *C. vulgaris* during the four days of growth. For instance, Eustance et al. [41] found that efficient nitrogen removal was caused by increased availability of dissolved inorganic carbon (DIC) at pH between 6 and 8. Conversely, because of the pH shifts observed with acetate and glucose (from 7.5 to 8.86 and 5.58, respectively), *C. vulgaris* cells could not have been actively taking up NH_4_^+^. In this context, a high NH_4_^+^ concentration in a medium with a pH below 6.5 causes a failure in the pH regulation mechanisms [41]. Still, in media with higher pH ranges (>7.7 at 25 °C), NH_4_^+^ could be toxic because of increases in free ammonia, which results in growth inhibition [42]. The removal of phosphate was found to be a much slower process than that observed for nitrogen. *C. vulgaris* showed the highest P-consumption rate with acetate: 14%, 1%, and 4% were removed for acetate, glucose, and glycerol, respectively.

As summarized in Figure 2, the effects of acetate, glucose, and glycerol on the fatty acid content of *C. vulgaris* under mixotrophic conditions were significant. The lipid productivities obtained with acetate, glucose, and glycerol were 29.17, 15.42, and 22.08 mg L^−1^ day^−1^, respectively. Heredia-Arroyo et al. [31] reported the maximum lipid productivity of *C. vulgaris* with glycerol as high as 160 mg L^–1^ d^–1^ with biomass productivities of 0.390 g L^−1^ d^−1^ after 72 h. Fatty acid composition profiles in mixotrophic *C. vulgaris* in acetate, glucose, and glycerol cultivation are dominated by three fatty acids: palmitic acid (C16:0), oleic acid (C18:1), and linoleic acid (C18:2). These fatty acids make up 65–85% of the fatty acid content under the tested conditions.

Finally, the growth of *C. vulgaris* using urea as a nitrogen source and glycerol as a carbon source is surprisingly different from that in other mixotrophic systems without external CO_2_ supply. The pH stability is an important characteristic that requires further research.

## 5. Conclusions

This study investigated the mixotrophic growth of *Chlorella vulgaris* with urea as a nitrogen source in the presence of MgSO_4_·7H_2_O. The response surface methodology showed that glycerol consumption is strongly influenced by MgSO_4_·7H_2_O concentration in the medium. Using the optimal urea and MgSO_4_·7H_2_O concentrations for glycerol consumption, it was possible to obtain 10% lipids on day four of the culture with a variety of sources of organic carbon (acetate, glucose, glycerol) in the medium; however, the lipid profile was highly affected by the organic carbon source. Furthermore, there was pH acidification in the presence of glucose, while in the presence of acetate, there was pH alkalinization. Unlike the pH variation with acetate and glucose, *C. vulgaris* kept the pH in the optimal range when glycerol was the carbon source. We conclude that:

- When glycerol is used as the carbon source, the optimal levels of urea and MgSO4·7H_2_O for *Chlorella vulgaris* culture under mixotrophic conditions are 1.7 g L^−1^ and 1.0 g L^−1^, respectively.
- At the optimal urea and MgSO_4_·7H_2_O concentrations for glycerol consumption, *C. vulgaris* consumed 3.8 g L^−1^ glycerol and 0.89 g L^−1^ urea, with a biomass production of 1.4 g L^−1^ d^−1^ after four days of growth.
- The medium’s carbon source (glycerol, glucose, or acetate) did not affect lipid production when the optimal urea and MgSO_4_·7H_2_O conditions were used; in this case, the average lipid production was 10%.
- Under the optimal growth conditions for glycerol consumption, the carbon source highly affected the lipid acid profile. With acetate, the major fatty acid was oleic acid (C18:1); followed by linoleic acid (C18:2); with glucose and glycerol, the profile was dominated by linoleic acid (C18:2) and palmitic acid (C16:0)
- The carbon source also affected the bulk solution’s pH variation over time. Glucose acidified the medium, and acetate alkalinized it. However, glycerol maintained the pH at the most suitable value for *C. vulgaris* growth.
- The nitrogen removal from the liquid medium was 36%, 9%, and 7% when glycerol, acetate, and glucose were used as the carbon source.

## Figures and Tables

**Table 2:** CCD matrix of experimental runs and their observed and predicted glycerol consumption rates.

Coded Values			Real Values		Observed	Predicted
Run	X1	X2	X1-Urea (g L^−1^)	X2-MgSO_4_·7H_2_O (g L^−1^)	Glycerol Consumption Rate (mg L^−1^ d^−1^)	Glycerol Consumption Rate (mg L^−1^ d^−1^)
1	−1	−1	0.5	0.5	548.01	547.65
2	+1	−1	1.5	0.5	339.70	403.13
3	−1	+1	0.5	1.5	276.49	223.03
4	+1	+1	1.5	1.5	739.48	749.80
5	−1.41	0	0.3	1.0	409.73	449.85
6	+1.41	0	1.7	1.0	770.23	720.15
7	0	−1.41	1.0	0.3	411.53	369.01
8	0	+1.41	1.0	1.7	352.04	384.61
9	0	0	1.0	1.0	531.91	525.30
10	0	0	1.0	1.0	578.12	525.30
11	0	0	1.0	1.0	555.08	525.30
12	0	0	1.0	1.0	461.41	525.30
13	0	0	1.0	1.0	500.00	525.30

**Table 3:** ANOVA of regression parameters of the response surface quadratic models of glycerol consumption rate.

Source	Sum of Squares	df	Mean Square	F-Value	*p*-Value
Model	2.35 × 10^−5^	5	47,074.16	14.70	0.0014 *
X_1_	73,058.06	1	73,058.06	22.81	0.002 *
X_2_	243.36	1	243.36	0.076	0.7908
X_1×2_	1.13 × 10^−5^	1	1.13 × 10^−5^	35.18	0.0006 *
X_1_^2^	6197.65	1	6197.65	1.94	0.2068
X_2_^2^	38,350.52	1	38,350.52	11.98	0.0105 *
Residual	22,416.54	7	3202.36		
Lack of Fit	13,974.43	3	4658.14	2.21	0.2297
Pure Error	8442.11	4	2110.53		
Cor Total	2.58 × 10^−5^	12			

**Table 4:** Point predicted solution by the model and verification of the model.

Solution 1 of 1 Response	Urea (g L^−1^)	MgSO4·7H_2_O (g L^−1^)	Predicted Mean	Experimentally Observed Glycerol Consumption Rate
Glycerol Consumption Rate (mg L^−1^ d^−1^)	1.5	1.0	718.82	723.52 ± 48.60

**Table 5:** Effects of organic carbon source (glycerol, acetate, or glucose) on the urea, nitrogen, and phosphorus consumption rates of *C. vulgaris* obtained at day 4.

Carbon Source	Biomass Production Rate (g DW L^−1^ d^−1^)	Fatty Acid Content (%)	pH Final	Urea Consumption (mg L^−1^ d^−1^)	Nitrogen (mg L^−1^ d^−1^)	Phosphorus (mg L^−1^ d^−1^)
Acetate	1.03 ± 0.05	11% ± 0.03	8.86 ± 0.01	144.60 ± 25.1	17.82 ± 0.38	14.75 ± 1.58
Glucose	0.70 ± 0.08	9.0% ± 0.06	5.68 ± 0.01	113.50 ± 16.7	14.64 ± 0.24	1.25 ± 1.189
Glycerol	0.88 ± 0.07	10% ± 0.04	6.88 ± 0.01	223.58 ± 6.8	71.27 ± 0.25	4.53 ± 1.11

## Data Availability

Data are available on request from the authors.

## References

[1] Shan S., Manyakhin A.Y., Wang C., Ge B., Han J., Zhang X., Zhou C., Yan X., Ruan R., Cheng P. (2023). Mixotrophy, a more promising culture mode: Multi-faceted elaboration of carbon and energy metabolism mechanisms to optimize microalgae culture. Bioresour. Technol..

[2] Yu H.-C., Lay C.-H., Abdul P.M., Wu J.-Y. (2023). Enhancing Lipid Production of *Chlorella* sp. by Mixotrophic Cultivation Optimization. Processes.

[3] Poddar N., Sen R., Martin G.J.O. (2018). Glycerol and nitrate utilisation by marine microalgae Nannochloropsis salina and *Chlorella* sp. and associated bacteria during mixotrophic and heterotrophic growth. Algal Res..

[4] Mohsenpour S.F., Hennige S., Willoughby N., Adeloye A., Gutierrez T. (2021). Integrating micro-algae into wastewater treatment: A review. Sci. Total Environ..

[5] Elsayed M., Eraky M., Osman A.I., Wang J., Farghali M., Rashwan A.K., Yacoub I.H., Hanelt D., Abomohra A. (2023). Sustainable valorization of waste glycerol into bioethanol and biodiesel through biocircular approaches: A review. Environ. Chem. Lett..

[6] Koley S., Sonkar S., Bagchi S.K., Patnaik R., Mallick N. (2022). Development of a low-cost cultivation medium for simultaneous production of biodiesel and bio-crude from the chlorophycean microalga Tetradesmus obliquus: A renewable energy prospective. J. Clean. Prod..

[7] Cao M., Kang J., Gao Y., Wang X., Pan X., Liu P. (2020). Optimization of cultivation conditions for enhancing biomass, polysaccharide and protein yields of Chlorella sorokiniana by response surface methodology. Aquac. Res..

[8] Vimali E., Senthil Kumar A., Sakthi Vignesh N., Ashokkumar B., Dhakshinamoorthy A., Udayan A., Arumugam M., Pugazhendhi A., Varalakshmi P. (2022). Enhancement of lipid accumulation in microalga *Desmodesmus* sp. VV2: Response Surface Methodology and Artificial Neural Network modeling for biodiesel production. Chemosphere.

[9] Solomon C.M., Collier J.L., Berg G.M., Glibert P.M. (2010). Role of urea in microbial metabolism in aquatic systems: A biochemical and molecular review. Aquat. Microb. Ecol..

[10] Syrett P., Rogers L.J., Gallon J.R. (1988). Uptake and utilization of nitrogen compounds. Biochemistry of the Algae and Cyanobacteria.

[11] Hodson R.C., Thompson J.F. (1969). Metabolism of Urea by Chlorella vulgaris. Plant Physiol..

[12] Hodson R.C., Williams S.K., Davidson W.R. (1975). Metabolic Control of Urea Catabolism in Chlamydomonas reinhardi and Chlorella pyrenoidosa. J. Bacteriol..

[13] Kanamori T., Kanou N., Kusakabe S., Atomi H., Imanaka T. (2005). Allophanate hydrolase of Oleomonas sagaranensis involved in an ATP-dependent degradation pathway specific to urea. FEMS Microbiol. Lett..

[14] Hermans C., Verbruggen N. (2005). Physiological characterization of Mg deficiency in Arabidopsis thaliana. J. Exp. Bot..

[15] Pasternak K., Kocot J., Horecka A. (2010). Biochemistry of magnesium. J. Elem..

[16] Rincon S.M., Romero H.M., Aframehr W.M., Beyenal H. (2017). Biomass production in Chlorella vulgaris biofilm cultivated under mixotrophic growth conditions. Algal Res..

[17] Kong W.B., Yang H., Cao Y.T., Song H., Hua S.F., Xia C.G. (2013). Effect of Glycerol and Glucose on the Enhancement of Biomass, Lipid and Soluble Carbohydrate Production by Chlorella vulgaris in Mixotrophic Culture. Food Technol. Biotechnol..

[18] Gautam K., Pareek A., Sharma D.K. (2013). Biochemical composition of green alga Chlorella minutissima in mixotrophic cultures under the effect of different carbon sources. J. Biosci. Bioeng..

[19] Rincon S.M., Urrego N.F., Avila K.J., Romero H.M., Beyenal H. (2019). Photosynthetic activity assessment in mixotrophically cultured Chlorella vulgaris biofilms at various developmental stages. Algal Res..

[20] Perez-Garcia O., Bashan Y. (2015). Microalgal Heterotrophic and Mixotrophic Culturing for Bio-refining: From Metabolic Routes to Techno-economics. Algal Biorefineries: Volume 2: Products and Refinery Design.

[21] Perez-Garcia O., Escalante F.M.E., de-Bashan L.E., Bashan Y. (2011). Heterotrophic cultures of microalgae: Metabolism and potential products. Water Res..

[22] Choi H.-J., Yu S.-W. (2015). Influence of crude glycerol on the biomass and lipid content of microalgae. Biotechnol. Biotechnol. Equip..

[23] Sun Y., Liu J., Xie T., Xiong X., Liu W., Liang B., Zhang Y. (2014). Enhanced Lipid Accumulation byChlorella vulgarisin a Two-Stage Fed-Batch Culture with Glycerol. Energy Fuels.

[24] Bo Y., Chu R., Sun D., Deng X., Zhou C., Yan X., Ruan R., Cheng P. (2023). Mixotrophic culture of bait microalgae for biomass and nutrients accumulation and their synergistic carbon metabolism. Bioresour. Technol..

[25] Paranjape K., Leite G.B., Hallenbeck P.C. (2016). Strain variation in microalgal lipid production during mixotrophic growth with glycerol. Bioresour. Technol..

[26] Yang C., Hua Q., Shimizu K. (2000). Energetics and carbon metabolism during growth of microalgal cells under photoautotrophic, mixotrophic and cyclic light-autotrophic/dark-heterotrophic conditions. Biochem. Eng. J..

[27] Sharma A.K., Sahoo P.K., Singhal S., Patel A. (2016). Impact of various media and organic carbon sources on biofuel production potential from *Chlorella* spp.. 3 Biotech.

[28] Skorupskaite V., Makareviciene V., Levisauskas D. (2015). Optimization of mixotrophic cultivation of microalgae *Chlorella* sp. for biofuel production using response surface methodology. Algal Res..

[29] Andruleviciute V., Makareviciene V., Skorupskaite V., Gumbyte M. (2014). Biomass and oil content of *Chlorella* sp., *Haematococcus* sp., *Nannochloris* sp. and *Scenedesmus* sp. under mixotrophic growth conditions in the presence of technical glycerol. J. Appl. Phycol..

[30] Liang Y.N., Sarkany N., Cui Y. (2009). Biomass and lipid productivities of Chlorella vulgaris under autotrophic, heterotrophic and mixotrophic growth conditions. Biotechnol. Lett..

[31] Heredia-Arroyo T., Wei W., Ruan R., Hu B. (2011). Mixotrophic cultivation of Chlorella vulgaris and its potential application for the oil accumulation from non-sugar materials. Biomass Bioenergy.

[32] Abomohra A., Li M., Faisal S., Li L., Elsayed M. (2022). Maximizing Nitrogen Removal and Lipid Production by Microalgae under Mixotrophic Growth Using Response Surface Methodology: Towards Enhanced Biodiesel Production. Fermentation.

[33] Khuri A.I. (2017). Response Surface Methodology and Its Applications in Agricultural and Food Sciences. Biom. Biostat. Int. J..

[34] (2010). I. Water Quality—Determination of Total Nitrogen after UV Digestion—Method Using Flow Analysis (CFA and FIA) and Spectrometric Detection.

[35] Dafner E.V., Szmant A.M. (2014). A modified segmented continuous flow analysis method for simultaneous determination of total dissolved nitrogen and phosphorus in marine environments. Limnol. Oceanogr. Methods.

[36] Clark S., Francis P.S., Conlan X.A., Barnett N.W. (2007). Determination of urea using high-performance liquid chromatography with fluorescence detection after automated derivatisation with xanthydrol. J. Chromatogr. A.

[37] Frieler R.A., Mitteness D.J., Golovko M.Y., Gienger H.M., Rosenberger T.A. (2009). Quantitative determination of free glycerol and myo-inositol from plasma and tissue by high-performance liquid chromatography. J. Chromatogr. B Anal. Technol. Biomed. Life Sci..

[38] Glibert P.M., Wilkerson F.P., Dugdale R.C., Raven J.A., Dupont C.L., Leavitt P.R., Parker A.E., Burkholder J.M., Kana T.M. (2016). Pluses and minuses of ammonium and nitrate uptake and assimilation by phytoplankton and implications for productivity and community composition, with emphasis on nitrogen-enriched conditions. Limnol. Oceanogr..

[39] Wang J., Curtis W.R. (2015). Proton stoichiometric imbalance during algae photosynthetic growth on various nitrogen sources: Toward metabolic pH control. J. Appl. Phycol..

[40] Barros A., Guerra L.T., Simões M., Santos E., Fonseca D., Silva J., Costa L., Navalho J. (2017). Mass balance analysis of carbon and nitrogen in industrial scale mixotrophic microalgae cultures. Algal Res..

[41] Eustance E., Gardner R.D., Moll K.M., Menicucci J., Gerlach R., Peyton B.M. (2013). Growth, nitrogen utilization and biodiesel potential for two chlorophytes grown on ammonium, nitrate or urea. J. Appl. Phycol..

[42] Vasconcelos Fernandes T., Shrestha R., Sui Y., Papini G., Zeeman G., Vet L.E., Wijffels R.H., Lamers P. (2015). Closing Domestic Nutrient Cycles Using Microalgae. Environ. Sci. Technol..

